# Inverse association of plasma hydrogen sulfide levels with visceral fat area among Chinese young men: a cross-sectional study

**DOI:** 10.20945/2359-3997000000339

**Published:** 2021-03-19

**Authors:** Dongmei Fan, Huiyan Huang, Xing Wang, Junru Liu, Bowei Liu, Fuzai Yin

**Affiliations:** 1 The First Hospital of Qinhuangdao Department of Endocrinology Qinhuangdao China Department of Endocrinology, The First Hospital of Qinhuangdao, Qinhuangdao, China.; 2 Dalian Hospital affiliated to Shengjing Hospital of China Medical University Department of Endocrinology Shenyang China Department of Endocrinology, Dalian Hospital affiliated to Shengjing Hospital of China Medical University, Shenyang, China.

**Keywords:** Hydrogen sulfide, visceral fat, correlation study, bioelectrical impedance, free fatty acids

## Abstract

**Objective:**

To investigate the association between plasma Hydrogen Sulfide (H_2_S) levels and visceral fat area (VFA) among Chinese young men.

**Subjects and methods:**

This cross-sectional study involved 156 Chinese male subjects, aged 18–45 years, who visited the First Hospital of Qinhuangdao (Hebei, China) in 2014 for annual health check-up. Participants were categorized into: low (VFA < 75.57 cm^2^), medium (75.57 cm^2^ ≤ VFA<100.37 cm^2^), and high (VFA ≥ 100.37 cm^2^) (n = 52/group). We estimated VFA and plasma H_2_S levels by using bioelectrical impedance analysis and a fluorescence probe-based approach, respectively. The associations of H_2_S with VFA and obesity anthropometric measures were assessed.

**Results:**

In the high VFA group, the body mass index (BMI, 30.4 ± 2.45 kg/m^2^), total body fat (TBF, 27.9 ± 3.23 kg), plasma H_2_S (3.5 μmol/L), free fatty acid (FFA, 0.6 ± 0.24 mmol/L), triglyceride (TG, 2.0 mmol/L), and total cholesterol (TC, 5.5 ± 1.02 mmol/L) levels were significantly higher than that of those of the low and medium VFA groups, respectively (P < 0.05). Plasma H_2_S levels were found to be inversely correlated with VFA, TBF, waist circumference, BMI, FFA, LnFINS, LnHOMA-IR, LnTG, TC, and LDL-C (*P* < 0.05). Multiple backward stepwise regression analysis revealed an inverse correlation of plasma H_2_S levels with FFA (β = −0.214, P = 0.005) and VFA (β = −0.429, P < 0.001), independent of adiposity measures and other confounding factors.

**Conclusion:**

VFA was independently and inversely associated with plasma H_2_S levels among Chinese young men. Therefore, determining plasma H_2_S levels could aid in the assessment of abnormal VAT distribution.

## INTRODUCTION

It is well-known that the fat tissue in our body is distributed into two main compartments – subcutaneous adipose tissue (SAT) and visceral adipose tissue (VAT), each having distinct metabolic features (1). Owing to its ubiquitous involvement in different medical pathologies, most of the research has focused on visceral adiposity. Visceral obesity or central obesity, characterized by an excessive accumulation of VAT, has been linked to different pathologies including, metabolic syndromes, chronic inflammation, coronary artery disease (CAD), deranged glucose and lipid metabolism, insulin resistance (2,3), increased predisposition to cancers of the colon (4), breast (5), and prostate (6). Moreover, it has been shown to be associated with prolonged hospital stays, infections and non-infectious complications, and in-hospital mortality (7). Abnormal VAT accumulation predisposes an individual to ischemic heart disease, arterial hypertension, and/or comprehensive cardiovascular risk (3,8–10). The bulk of evidence suggests that visceral fat is the key mediator between the multiple facets of the metabolic syndrome: glucose intolerance, hypertension, dyslipidemia, and insulin resistance (11). However, due to the presence of metabolic heterogeneity among obese patients with similar levels of VAT, individual genetic susceptibility may be responsible in modulating the risk associated with the excessive accumulation of VAT (12). Owing to its potential association with worse prognosis, metabolic abnormalities, and degree of disease activity in various chronic diseases, it is necessary to quantify VAT from total adipose tissue.

To date, several techniques have been developed for measuring visceral adiposity ranging from simple, indirect methods of evaluation, such as body mass index (BMI), waist-to-hip circumference ratios (WHR), and waist circumference (WC) to imaging techniques, such as computed tomography (CT) that not only estimates the amount of abdominal visceral fat but also measures multi-compartment body fat (1,13,14). Anthropometric measures and bioelectrical impedance analysis (BIA) were developed to provide measures of body composition. BIA is an easily accessible, safe, and a cost-effective method for estimating body composition (15,16). Apart from measuring whole-body fat content, BIA determines fat-free mass. Significant correlations were observed when BIA was employed to assess the amount of abdominal subcutaneous and visceral fat, in comparison with precise imaging techniques such as CT (17,18).

Growing evidence sheds light on the multifaceted roles of hydrogen sulfide (H_2_S) in adipose tissue. Cystathionine γ lyase (CSE)-derived H_2_S expressed by adipocytes regulates several biological activities in adipose tissue, including inflammation, apoptosis, insulin resistance, adipokine secretion, and adipocyte differentiation (19). H_2_S, a novel endogenous gaseous signal transducer (gasotransmitter) is naturally synthesized by CSE, cystathionine β-synthetase (CBS), 3-mercaptopyruvate sulfur transferase (3-MST) and cysteine aminotransferase (CAT). Additionally, enzymatic production of H_2_S occurs *in vivo* from L-cysteine (LC) (20). Alterations in H_2_S levels or H_2_S synthetase expression have been implicated in the pathogenesis of many pathophysiological processes, such as neurological systems, vascular function, energy metabolism and biogenesis, obesity, and ageing (20). Consequently, in recent years, there has been a surge in research on adipose tissue-derived endogenous H_2_S and its pathophysiological roles in adipose tissue, with a focus related to its effects on adipose tissue inflammation, apoptosis, adipokine secretion, glucose and lipid metabolism, and vascular tension (21). Despite this, the complex role of H_2_S in the regulation of adipose tissue metabolism has not been fully understood. Several published data have highlighted the importance of H_2_S in the physiology and pathophysiology of the nervous, cardiovascular, and gastrointestinal systems via its antioxidant, anti-inflammatory (22), antinociceptive, antihypertensive, neuromodulative, and cytoprotective effects (23). In addition, beneficial roles of H_2_S in anti-apoptosis of cardiomyocytes and other cardiovascular processes have also been reported (24,25). Further, reduced plasma levels of H_2_S have been observed in patients with ischemia (26), diabetes (27), high-fat diet-induced cardiomyopathy (28), and hypertension (29).

A recent study has utilized computed tomography (CT) to evaluate visceral obesity by measuring visceral fat area (VFA), and reported significant associations between VFA and metabolic disturbances (9). However, studies investigating the relationship between plasma levels of H_2_S and VFA and obesity anthropometric measures are rare. Therefore, in view of the above, this study aimed to analyze the associations of plasma levels of H_2_S with VFA (measured by BIA) and obesity anthropometric measures among Chinese young men, and further speculated whether determining plasma H_2_S levels could aid in the assessment of abnormal VAT distribution.

## SUBJECTS AND METHODS

### Study design and subjects

This cross-sectional study involved 156 Chinese male subjects (N = 156), aged 18–45 years, who visited the First Hospital of Qinhuangdao (Hebei, China) in 2014 for annual health check-up and who had maintained a stable body weight (< 2.5 kg) for over 3 months prior to enrollment. Subjects with a previous medical history of diabetes, dyslipidemia, or coronary artery diseases, secondary obesity (hypophyseal tumor, hypothyroidism, or drug-induced obesity), uncontrolled hypertension (>160/90 mmHg), cardiovascular and/or peripheral vascular diseases, malignant tumors, severe hepatic or renal dysfunction (> 1.5-fold elevation of alanine aminotransferase and aspartate aminotransferase, or serum creatinine > 115 μmol/L), acute/chronic inflammation and/or fever were excluded from this study. Those who were current or former smokers (participants who have a smoking history equivalent to at least one cigarette per day for more than 6 months were defined as smokers), and/or were heavy drinkers (participants who consumed more than 80 g of alcohol at least once per day for two weeks or had been drinking more than 40 g of alcohol for over 5 years) were not included in this study. Further, we excluded those who underwent specific treatment for metabolic abnormalities (consumption of weight-loss products, in the context of medicine, health, or physical fitness), or those who took medications known to affect glucose and lipid metabolism, such as statins, glucocorticoids, thyroid hormones, and thiazide diuretics. Of note, in this study, participants were enrolled according to strict inclusion and exclusion criteria. This study was conducted according to the STROBE guidelines (30).

Participants were categorized into low (VFA < 75.57 cm^2^), medium (75.57 cm^2^ ≤ VFA<100.37 cm^2^), and high (VFA ≥ 100.37 cm^2^) VFA groups (n = 52/group). The cut-off values for VFA groups were determined based on tertile distribution. This study was approved by the ethics committee of the First Hospital of Qinhuangdao. Written informed consent was obtained from all subjects prior to enrollment.

### Blood sampling and plasma collection

After 10-hours overnight fasting, blood samples were collected from the antecubital vein into K2-EDTA tubes. Plasma specimens were obtained immediately after collection by centrifuging the samples for 10 min at 3,500 rpm at 4°C and were stored at −80°C until further use.

### Estimation of plasma H_2_S levels

Plasma H_2_S levels were measured by using a modified fluorescence probe-based approach, previously described by Wu and cols. (31). Briefly, 100 pmol of H_2_S sensitive probes in 20 μL ethanol were added in 96-well microplates by using an interlaced model in the plate. An equal volume of ethanol was also added into the uncoated wells. Subsequently, the plates were allowed to air dry in dark for 1 h and were then stored in a sealed condition at −20°C. Equal volumes (150 μL) of plasma sample and saturated ammonium sulfate buffer (pH 7.8) were mixed and then centrifuged at 25,000×g for 15 min at 4°C. The supernatant was transferred into a new tube and re-centrifuged. Subsequently, 100 μL of supernatant was added into the probe-coated and uncoated wells, respectively. Following incubation in a dark environment at 37°C for 2 h, the fluorescence intensity in each well was acquired with excitation at λEX (340 nM) and emission at λEM (445 nM) by using a FLUOstar^®^ OPTIMA microplate reader (BMG Labtech, Ortenberg, Germany). The discrepant fluorescence intensity values between the coated and uncoated wells were measured, and the corresponding plasma H_2_S concentrations were determined on the basis of standard calibration curves constructed with several known H_2_S concentrations and sodium hydrogen sulfide (NaHS).

### Measurement data

Physical measurements of height and weight of each subject were obtained by using an electronic digital scale (HGM-800, Henan Shengyuan Industrial Co., Ltd.). During all measurements, subjects wore light clothing and were barefoot. The WC was measured from the front at the mid-point between the rib cage and the lateral iliac crest after full expiration, while the subject was breathing gently. Electronic sphygmomanometer (HBP-9020; Omron, Osaka, Japan) was used to record blood pressure levels [systolic blood pressure (SBP); diastolic blood pressure (DBP)] of seated participants. Two consecutive readings were taken 10 min apart and the average was used for analysis. BMI was calculated as weight (kg) divided by square of height (m^2^).

A bioelectrical impedance analysis (BIA) device, InBody S10 (Inbody Co., Ltd., Seoul, Korea) was used to measure total body fat (TBF) and VFA. All measurements were performed on subjects in seated position. Typically, this device includes eight electrodes and uses thumb and middle finger of both the hands and both feet. The whole process took about 5 min.

Levels of fasting blood glucose (FBG), plasma free fatty acids (FFA), and plasma lipids (total cholesterol (TC), total triglycerides (TG), low-density lipoprotein cholesterol (LDL-C), and high-density lipoprotein cholesterol (HDL-C) were measured by using the glucose oxidase-phenol 4-aminoantipyrine peroxidase method, and enzymatic colorimetric assays with a biochemical auto-analyzer (Hitachi 7600 automated analyzer, Tokyo, Japan). Fasting insulin concentration (FINS) was measured by using an enzyme linked immunosorbent assay (ELISA) kit (intra-assay coefficient of variation (CV%) < 3 and inter-assay CV% < 4; USCN Life Science Inc., USA) on a microplate reader (Model 680; Bio-Rad, USA). The Homeostatic Model Assessment of Insulin Resistance (HOMA-IR) index was computed as follows: [fasting insulin level (mIU/L) × fasting glucose level (mmol/L)]/22.5.

### Statistical analyses

All analyses were performed by using the SPSS statistical software (SPSS for Windows, Version 14.0 SPSS Inc., Chicago, IL, USA). Data were expressed as mean ± standard deviation. Non-normally distributed data were presented as median and interquartile range (IQR) and were analyzed after logarithmic transformation. Differences between groups were evaluated by using analysis of variance (ANOVA) and Pearson's Chi-Square test. Multiple comparisons with statistically significant variables were performed by using least significant difference (LSD) test. The association of plasma H_2_S levels with various parameters was determined by using multiple backward stepwise linear regression models, with plasma H_2_S levels as the dependent variable, and age, VFA, TBF, WC, BMI, SBP, DBP, FFA, FINS, HOMA-IR, TG, TC, and LDL-C as independent variables. *P* < 0.05 was considered statistically significant.

## RESULTS

### Characteristics of the study population

A total of 156 male subjects (N =156) were included in our analyses. There were 52 subjects (n = 52) in each of the three groups (low, medium, and high VFA groups). The mean ages of subjects in low, medium, and high VFA groups were 35.6 ± 6.02, 36.2 ± 6.19, and 34.5 ± 6.41 years, respectively, while the mean weights were 72.4 ± 6.62, 83.4 ± 6.71, and 96.1 ± 10.81 kgs, respectively. There were no significant differences in age (*P* = 0.345) and DBP (*P* = 0.105). The anthropometric measures, BMI (30.4 ± 2.45 kg/m^2^), and WC (102.2 ± 6.12 cm) of the high VFA group were significantly higher than those of the low and medium VFA groups, respectively (*P* < 0.05). Similarly, in the high VFA group, TBF (27.9 ± 3.23 kg), plasma H_2_S (3.5 μmol/L), FFA (0.6 ± 0.24 mmol/L), FBG (5.9 ± 1.09 mmol/L), FINS (12.9 uIU/mL), TG (2.02 mmol/L), TC (5.5 ± 1.02 mmol/L), HDL-C (1.1 ± 0.24 mmol/L), and LDL-C (3.8 ± 1.10 mmol/L) levels and HOMA-IR (3.4) were significantly higher than those of the low and medium VFA groups, respectively (*P* < 0.05). Table 1 summarizes clinical and laboratory characteristics of Chinese male subjects in the three VFA groups.

**Table 1:** Clinical and laboratory characteristics of Chinese male subjects in different groups

Characteristic variable	VFA			P value
	Low <75.57 cm^2^ (n = 52)	Medium 75.57-100.37 cm^2^ (n = 52)	High ≥100.37 cm^2^ (n = 52)	
Age (years)	35.6 ± 6.02	36.2 ± 6.19	34.5 ± 6.41	0.345
Weight (kg)	72.4 ± 6.62	83.4 ± 6.71	96.1 ± 10.81	<0.05*
TBF (kg)	15.3 ± 2.93	21.9 ± 2.43#	27.9 ± 3.23#^§^	<0.05*
WC (cm)	84.3 ± 7.98	95.8 ± 4.73#	102.2 ± 6.12#^§^	<0.05*
BMI (kg/m^2^)	23.8 ± 2.42	27.8 ± 1.96#	30.4 ± 2.45#^§^	<0.05*
SBP (mmHg)	123.1 ± 13.71	129.9 ± 13.13#	130 ± 13.12#	0.012*
DBP (mmHg)	81.3 ± 9.59	83.7 ± 10.94	85.6 ± 9.78	0.105
H*2*S (μmol/L)	4.9 (4.08, 6.15)	3.9 (3.30, 4.18)#	3.5 (2.91, 3.96)#^§^	<0.05*
FFA (mmol/L)	0.4 ± 0.16	0.5 ± 0.19#	0.6 ± 0.24#^§^	0.012*
FBG (mmol/L)	5.5 ± 0.51	5.5 ± 0.56	5.9 ± 1.09#^§^	0.005*
FINS (uIU/mL)	8.8 (7.54, 11.51)	10.6 (9.04, 13.71)	12.9 (9.11, 17.84)#^§^	0.001*
HOMA-IR	2.2 (1.74, 3.00)	2.7 (2.21, 3.36)	3.4 (2.36, 4.81)#^§^	<0.05*
TG (mmol/L)	1.2 (0.96, 2.09)	1.6 (1.12, 2.71)#	2.0 (1.42, 2.75)#	<0.05*
TC (mmol/L)	4.9 ± 0.72	5.2 ± 1.12	5.5 ± 1.02#	0.004*
HDL-c (mmol/L)	1.2 ± 0.30	1.1 ± 0.23#	1.1 ± 0.24#	0.001*
LDL-c (mmol/L)	3.0 ± 0.77	3.5 ± 0.90#	3.8 ± 1.10#	<0.05*

VFA: Visceral fat area; TBF: total body fat; WC: waist circumference; BMI: body mass index; SBP: systolic blood pressure; DBP: diastolic blood pressure; H*2*S: hydrogen sulfide; FFA: free fatty acid; FBG: fasting blood glucose; FINS: fasting insulin; HOMA-IR: Homeostatic Model Assessment of Insulin Resistance; TG: triglyceride; TC: total cholesterol; HDL-c: high density lipoprotein-cholesterol; LDL-c: Low density lipoprotein-cholesterol.

Normally distributed variables were expressed as means ± SD. Non-normally distributed variables were expressed as medians (inter-quartile range, IQR). Comparisons were done between the three groups using analysis of variance (ANOVA). Multiple comparisons with statistically significant variables were performed by using least significant difference (LSD) test.

* *P* < 0.05 was considered significant.

# Compared with low VFA group. §Compared with medium VFA group.

### Univariate regression analysis

Univariate analysis was performed to analyze the association of plasma H_2_S levels with anthropometric indices of obesity and other laboratory parameters (Table 2). Plasma H_2_S levels were found to be negatively correlated with VFA (r = −0.502, *P* < 0.05), TBF (r = −0.403, *P* < 0.05), WC (r = −0.430, *P* < 0.05), BMI (r = −0.460, *P* < 0.05), FFA (r = −0.298, *P* < 0.05), LnFINS (r = −0.283, *P* = 0.003), LnHOMA-IR (r = −0.240, *P* = 0.003), LnTG (r = −0.207, *P* = 0.009), TC (r = −0.221, *P* = 0.006) and LDL-C (r = −0.289, *P* < 0.05).

**Table 2:** Univariate analysis of correlations between plasma H*2*S levels and other variables

Variable	r	*P* value
Age (years)	-0.045	0.576
VFA (cm^2^)	-0.502	<0.05*
TBF (kg)	-0.403	<0.05*
WC (cm)	-0.430	<0.05*
BMI (kg/m^2^)	-0.460	<0.05*
SBP (mmHg)	-0.154	0.056
DBP (mmHg)	-0.157	0.051
FFA (mmol/L)	-0.298	<0.05*
FBG (mmol/L)	-0.071	0.377
LnFINS	-0.283	0.003*
LnHOMA-IR	-0.240	0.003*
LnTG	-0.207	0.009*
TC (mmol/L)	-0.221	0.006*
HDL-C (mmol/L)	0.074	0.391
LDL-C (mmol/L)	-0.289	<0.05*

H_2_S: hydrogen sulfide; VFA: visceral fat area; TBF: total body fat; WC: waist circumference; BMI: body mass index; SBP: systolic blood pressure; DBP: diastolic blood pressure; FFA: free fatty acid; FBG: fasting blood glucose; LnFINS: the natural logarithm (Ln) of fasting insulin; LnHOMA-IR: the natural logarithm of Homeostatic Model Assessment of Insulin Resistance; LnTG: the natural logarithm of triglyceride; TC: total cholesterol; HDL-C: high density lipoprotein-cholesterol; LDL-C: low density lipoprotein-cholesterol.

Non-normally distributed variables were analyzed after logarithmic transformation.

* *P* < 0.05 was considered significant.

### Multiple linear regression analysis

Multiple backward stepwise regression analysis revealed that plasma H_2_S levels were independently and inversely associated with FFA (β = −0.214, *P* = 0.005) and VFA (β = −0.429, *P* < 0.001) after adjusting for age, BMI, WC, TBF, FINS, HOMA-IR, TG, TC, and LDL-C (Table 3).

**Table 3:** Multiple linear regression analysis

Variables	Unadjusted β-coefficient	S.E.	β-coefficient^a^	t	*P* value	*r* ^2^
VFA	-0.006	0.001	-0.429	-5.685	<0.001*	0.256
FFA	-0.382	0.135	-0.214	-2.834	0.005*	0.040

VFA: visceral fat area; FFA: free fatty acid; S.E: standard error. Unstandardized β-coefficients were derived from multivariate linear regression.

a The estimates were adjusted for the confounding effects of age, BMI (body mass index), WC (waist circumference), TBF (total body fat), FINS (fasting insulin), HOMA-IR (Homeostatic Model Assessment of Insulin Resistance), TG (triglyceride), TC (total cholesterol), and LDL-C (low density lipoprotein-cholesterol).

* *P* < 0.05 was considered significant.

## DISCUSSION

Our study reveals independent, inverse associations of plasma H_2_S levels with VFA and FFA among Chinese young men, and further demonstrated that plasma H_2_S levels were negatively correlated with VFA, TBF, WC, BMI, FFA, LnFINS, LnHOMA-IR, LnTG, TC, and LDL-C.

Adipose tissue is one of the largest, complex endocrine organs that secretes a variety of factors (32), which play a significant role in the development of systemic oxidative stress (33). Recent studies have demonstrated that H_2_S is synthesized by cystathionine γ-lyase CSE in perivascular adipose tissue (PVAT) (34). VAT is a hormonally active component of TBF and its abnormal high deposition leads to visceral obesity (1). VAT has been associated with the metabolic consequences of obesity (35). Further, in obese patients, VAT has been shown to abnormally release adipokines and FFAs (36), thus promoting systemic oxidative stress. In contrast to subcutaneous adipocytes, visceral adipocytes are characterized by a hyperlipolytic profile, and individuals with more VAT tend to have a high concentration of circulating FFAs (37), which is consistent with the finding of this study. It has been hypothesized that visceral fat is largely a VAT marker for excess FFA release. Therefore, metabolic abnormalities resulting as a consequence of increased visceral adiposity may be due to the exposure of lean tissues to high FFA concentrations (38). Of note, high FFA levels have been shown to stimulate the production of reactive oxygen species (ROS), including hydroxyl radicals, superoxide anions, and hydrogen peroxide (H_2_O_2_) in the endothelial and vascular smooth muscle cells (39), thus generating high levels of H_2_O_2_ in the mitochondria (40). On the contrary, endogenous H_2_S, which has the ability to scavenge ROS, protects the cells against any oxidative damage (41). Therefore, during stress, there is a high consumption of endogenous H_2_S for the elimination of excessive ROS, which may lead to decreased plasma H_2_S levels in individuals with high FFA. This hypothesis is further supported by the independent inverse correlation of plasma H_2_S levels with FFA demonstrated in this study. Taken together, these findings indicate that plasma H_2_S levels may reflect VAT distribution and FFA metabolism. Delving further, low plasma H_2_S levels have been found in patients with type 2 diabetes or obesity, among other metabolic derangements (19).

It has been reported that adiposity rather than diabetes status is a major determinant of plasma H_2_S levels (42), which highlights the role of H_2_S metabolism in obesity. Consistent with a previous study, adiposity measures (WC, TBF, and BMI) showed a negative correlation with plasma H_2_S levels among Chinese young men, which implies the existence of an inverse relationship between the degree of obesity and H_2_S production (42). Further lending strength to this finding, a previous study has demonstrated that plasma H_2_S levels were lowest in subjects with obesity and type 2 diabetes, and suggested that adiposity may be the key driving force for predicting low plasma H_2_S levels (43). Another study showed that adipose tissue-derived low levels of endogenous H_2_S may contribute to adipose tissue inflammation associated with obesity/metabolic syndrome, or conversely excess H_2_S levels might result in insulin resistance in metabolic syndrome (21).

Our study is the first-of-its-kind to demonstrate an inverse correlation between VFA and plasma H_2_S levels, independent of adiposity measures and other confounding factors. BMI and WC are the most commonly used central obesity anthropometric measures for assessing adiposity-related risk and body fat distribution (44). Despite the fact that WC reflects visceral and subcutaneous fat of VAT, VFA showed a strong, inverse association with plasma H_2_S levels, even after accounting for BMI and WC. Our finding is in line with a previous study which showed that VAT area, but not WC, was strongly associated with an unfavorable metabolic risk profile (3). As high VFA has been identified as a critical risk factor for metabolic syndromes and obesity-related complications, our observations further indicate that low plasma H_2_S levels might reflect the presence of metabolic abnormalities, and hence may be used as a potential early biomarker for metabolic syndromes and obesity-related diseases. In addition, reduced plasma H_2_S concentration was found to be associated with increased FINS, HOMA-IR, TG, TC, and LDL-C levels. However, these findings were not significant after adjusting for measures of adiposity (VFA, BMI, WC, and TBF). This could be possibly due to the inclusion of relatively younger subjects without any severe metabolic disorders. However, lack of independent correlations between plasma H_2_S levels and these indexes suggest that determining plasma H_2_S levels may not aid in the complete understanding of mechanisms through which H_2_S acts on adipose tissue metabolism, such as glycolipid metabolism. Interestingly, in this study, the association of plasma H_2_S levels with body fat distribution (TBF) shifted towards visceral fat accumulation, thus indicating that the visceral fat proportion of total body fat is more important for assessing the impact on metabolic disease (45). This finding is supported by a recent study, which showed that H_2_S reduced lipolysis of adipocytes in HFD mice without increasing total fat mass and body weight (19). However, the mechanism underlying the regulation of plasma H_2_S levels in presence of excess VFA remains elusive.

This study has a few limitations that should be considered when interpreting the results. As this was a cross-sectional study, the associations between VFA and plasma H_2_S levels cannot be construed as causal. Large population studies employing longitudinal methods would enable researchers to determine causal pathways and validate the directionality of this association. In addition, the heterogeneity of this association with respect to race, ethnicity, gender needs to evaluated. As this study design involved convenience sampling, and only male subjects aged between 18 and 45 years were recruited, generalization of our findings to other populations may be impeded. Further the lack of diversity among research participants may pose some limitation in the present study.

In conclusion, our study demonstrated that plasma H_2_S levels progressively declined (*P* < 0.05) in correlation with the degree of VFA among the three groups of Chinese young men. VFA was independently and inversely associated with plasma H_2_S levels among Chinese young men. Therefore, determining plasma H_2_S levels could aid in the assessment of abnormal VAT distribution.

## References

[1] Shuster A, Patlas M, Pinthus JH, Mourtzakis M (2012). The clinical importance of visceral adiposity: a critical review of methods for visceral adipose tissue analysis. Br J Radiol.

[2] Ritchie SA, Connell JM (2007). The link between abdominal obesity, metabolic syndrome and cardiovascular disease. Nutr Metab Cardiovasc Dis.

[3] Fox CS, Massaro JM, Hoffmann U, Pou KM, Maurovich-Horvat P, Liu CY (2007). Abdominal visceral and subcutaneous adipose tissue compartments: association with metabolic risk factors in the Framingham Heart Study. Circulation.

[4] Oh TH, Byeon JS, Myung SJ, Yang SK, Choi KS, Chung JW (2008). Visceral obesity as a risk factor for colorectal neoplasm. J Gastroenterol Hepatol.

[5] Schapira DV, Clark RA, Wolff PA, Jarrett AR, Kumar NB, Aziz NM (1994). Visceral obesity and breast cancer risk. Cancer.

[6] Von Hafe P, Pina F, Pérez A, Tavares M, Barros H (2004). Visceral fat accumulation as a risk factor for prostate cancer. Obes Res.

[7] Tsujinaka S, Konishi F, Kawamura YJ, Saito M, Tajima N, Tanaka O (2008). Visceral obesity predicts surgical outcomes after laparoscopic colectomy for sigmoid colon cancer. Dis Colon Rectum.

[8] Lamarche B, Lemieux S, Dagenais GR, Després JP (1998). Visceral obesity and the risk of ischaemic heart disease: insights from the Québec Cardiovascular Study. Growth Horm IGF Res.

[9] Ryo M, Kishida K, Nakamura T, Yoshizumi T, Funahashi T, Shimomura I (2014). Clinical significance of visceral adiposity assessed by computed tomography: A Japanese perspective. World J Radiol.

[10] Onat A, Avci GS, Barlan MM, Uyarel H, Uzunlar B, Sansoy V (2004). Measures of abdominal obesity assessed for visceral adiposity and relation to coronary risk. Int J Obes Relat Metab Disord.

[11] Desprès J-P, Angel A, Anderson H, Bouchard C, Lau D, Leiter L, Mendelson R (1996). Progress in Obesity Research: Proceedings of the Seventh International Congress on Obesity (Toronto, Canada, August 20–25, 1994).

[12] Desprès J-P, Moorjani S, Lupien PJ, Tremblay A, Nadeau A, Bouchard C (1992). Genetic aspects of susceptibility to obesity and related dyslipidemias. Mol Cell Biochem.

[13] Chowdhury B, Sjöström L, Alpsten M, Kostanty J, Kvist H, Löfgren R (1994). A multicompartment body composition technique based on computorized tomography. Int J Obes Relat Metab Disord.

[14] Sjöström L, Lönn L, Chowdhury B, Grangärd Lissner L, Sjöstrom D, Sullivan L, Angel A, Anderson H, Bouchard C, Lau D, Leiter L, Mendelson R (1996). Progress in Obesity Research: Proceedings of the Seventh International Congress on Obesity (Toronto, Canada, August 20–25, 1994).

[15] Mourtzakis M, Prado CM, Lieffers JR, Reiman T, McCargar LJ, Baracos VE (2008). A practical and precise approach to quantification of body composition in cancer patients using computed tomography images acquired during routine care. Appl Physiol Nutr Metab.

[16] Kyle UG, Bosaeus I, De Lorenzo AD, Deurenberg P, Elia M, Manuel Gómez J (2004). Bioelectrical impedance analysis-part II: utilization in clinical practice. Clin Nutr.

[17] Nagai M, Komiya H, Mori Y, Ohta T, Kasahara Y, Ikeda Y (2008). Development of a new method for estimating visceral fat area with multi-frequency bioelectrical impedance. Tohoku J Exp Med.

[18] Shoji K, Maeda K, Nakamura T, Funahashi T, Matsuzawa Y, Shimomura I (2008). Measurement of visceral fat by abdominal bioelectrical impedance analysis is beneficial in medical checkup. Obes Research Clin Pract.

[19] Zhu L, Yang B, Ma D, Wang L, Duan W (2020). Hydrogen Sulfide, Adipose Tissue and Diabetes Mellitus. Diabetes Metab Syndr Obes.

[20] Gheibi S, Samsonov AP, Gheibi S, Vazquez AB, Kashfi K (2020). Regulation of carbohydrate metabolism by nitric oxide and hydrogen sulfide: implications in diabetes. Biochem Pharmacol.

[21] Bełtowski J, Jamroz-Wiśniewska A (2016). Hydrogen sulfide in the adipose tissue-physiology, pathology and a target for pharmacotherapy. Molecules.

[22] Pan LL, Qin M, Liu XH, Zhu YZ (2017). The Role of Hydrogen Sulfide on Cardiovascular Homeostasis: An Overview with Update on Immunomodulation. Front Pharmacol.

[23] Lee SR, Nilius B, Han J (2018). Gaseous Signaling Molecules in Cardiovascular Function: From Mechanisms to Clinical Translation. Rev Physiol Biochem Pharmacol.

[24] Kashfi K, Olson KR (2013). Biology and therapeutic potential of hydrogen sulfide and hydrogen sulfide-releasing chimeras. Biochem Pharmacol.

[25] Bilban M, Haschemi A, Wegiel B, Chin BY, Wagner O, Otterbein LE (2008). Heme oxygenase and carbon monoxide initiate homeostatic signaling. J Mol Med (Berl).

[26] Shen Y, Shen Z, Miao L, Xin X, Lin S, Zhu Y (2015). miRNA-30 family inhibition protects against cardiac ischemic injury by regulating cystathionine-γ-lyase expression. Antioxid Redox Signal.

[27] Zhang L, Wang Y, Li Y, Li L, Xu S, Feng X (2018). Hydrogen Sulfide (H2S)-Releasing Compounds: Therapeutic Potential in Cardiovascular Diseases. Front Pharmacol.

[28] Barr LA, Shimizu Y, Lambert JP, Nicholson CK, Calvert JW (2015). Hydrogen sulfide attenuates high fat diet-induced cardiac dysfunction via the suppression of endoplasmic reticulum stress. Nitric Oxide.

[29] Sen U, Mishra PK, Tyagi N, Tyagi SC (2010). Homocysteine to hydrogen sulfide or hypertension. Cell Biochem Biophys.

[30] Vandenbroucke JP, von Elm E, Altman DG, Gøtzsche PC, Mulrow CD, Pocock SJ (2007). Strengthening the reporting of observational studies in epidemiology (STROBE): Explanation and elaboration. PLoS Med.

[31] Wu L, Yang W, Jia X, Yang G, Duridanova D, Cao K (2009). Pancreatic islet overproduction of H2S and suppressed insulin release in Zucker diabetic rats. Lab Invest.

[32] Pouliot M-C, Desprès J-P, Nadeau A, Moorjani S, Prud'Homme D, Lupien PJ (1992). Visceral obesity in men Associations with glucose tolerance, plasma insulin and lipoprotein levels. Diabetes.

[33] Desprès J-P, Moorjani S, Lupien PJ, Tremblay A, Nadeau A, Bouchard C (1990). Regional distribution of body fat, plasma lipoproteins and cardiovascular disease. Arteriosclerosis.

[34] Bełtowski J (2013). Endogenous hydrogen sulfide in perivascular adipose tissue: role in the regulation of vascular tone in physiology and pathology. Can J Physiol Pharmacol.

[35] Shen W, Punyanitya M, Wang Z, Gallagher D, St-Onge MP, Albu J (2004). Visceral adipose tissue: relations between single-slice areas and total volume. Am J Clin Nutr.

[36] Schernthaner GH, Schernthaner G (2005). Insulin resistance and inflammation in the early phase of type 2 diabetes: potential for therapeutic intervention. Scand J Clin Lab Invest Suppl.

[37] Gasteyger C, Tremblay A (2002). Metabolic impact of body fat distribution. J Endocrinol Invest.

[38] Ebbert JO, Jensen MD (2013). Fat depots, free fatty acids, and dyslipidemia. Nutrients.

[39] Inoguchi T, Li P, Umeda F, Yu HY, Kakimoto M, Imamura M (2000). High glucose level and free fatty acid stimulate reactive oxygen species production through protein kinase C--dependent activation of NAD(P)H oxidase in cultured vascular cells. Diabetes.

[40] Anderson EJ, Lustig ME, Boyle KE, Woodlief TL, Kane DA, Lin CT (2009). Mitochondrial H2O2 emission and cellular redox state link excess fat intake to insulin resistance in both rodents and humans. J Clin Invest.

[41] Kimura H (2014). Production and physiological effects of hydrogen sulfide. Antioxid Redox Signal.

[42] Whiteman M, Gooding KM, Whatmore JL, Ball CI, Mawson D, Skinner K (2010). Adiposity is a major determinant of plasma levels of the novel vasodilator hydrogen sulphide. Diabetologia.

[43] Carter RN, Morton NM (2016). Cysteine and hydrogen sulphide in the regulation of metabolism: insights from genetics and pharmacology. J Pathol.

[44] Park YS, Kim J-S (2012). Obesity phenotype and coronary heart disease risk as estimated by the Framingham risk score. J Korean Med Sci.

[45] Kim SK, Park SW, Kim SH, Cha BS, Lee HC, Cho YW (2009). Visceral fat amount is associated with carotid atherosclerosis even in type 2 diabetic men with a normal waist circumference. Int J Obesity.

